# Croup

**Published:** 1874-04

**Authors:** 


### CROUP.

THIS dangerous ailment can be alleviated in a few minutes and eventually cured by several domestic remedies, detailed in the family doctor book, entitled "HEALTH AT HOME," thus: Have three or four thicknesses of common flannel in a basin of ice water, apply it along the front and sides of the throat to an inch or two below the little hollow at the bottom of the neck; hold it on with a dry flannel; have another such in the basin to apply in three minutes; use them thus alternately until relief is obtained, known by easier breathing and looser phlegm. Hot water, as hot as can be borne, applied in the same way, is quite as efficacious. In both cases flannels should be pressed out just so as not to dribble and wet the clothing, and in every way the feet and whole body should be kept abundantly warm. Both methods act on the same principle, abstracting the extra heat and inflammation of the parts. Most cases of croup are caused by children being allowed to be out of doors, in the early spring and later fall, after sundown.

